# Supplementary material for: Steps on the Path to Clinical Translation—A British and Irish Chapter ISMRM Workshop Survey of the UK MRI Community
Source: Magn Reson Med. 2025 Dec 19;95(4):1934–43. doi: 10.1002/mrm.70225 (PMC12850563; doi:10.1002/mrm.70225)
Supplement: Supplementary file 2 — Data S2: mrm70225‐sup‐0002‐DataS2.docx. [file MRM-95-1934-s001.docx]

| **Survey questions** | **Answers** |
| --- | --- |
| **Q1**   1. **Is an imaging biomarker useful if it doesn't give a yes/no answer to aid diagnosis?** 2. **How can we standardise the language we use to aid clinical translation? Should effort be made to develop a consensus paper to standardise terminology to aid clinical translation?** 3. **Quantitative MR parameter maps often need to be created away from the scanner console. What is the maximum amount of time after acquisition, if an imaging biomarker is to be integrated into the clinical workflow?** | A. No **(5%)**, Yes **(83%)**, Unsure **(9%)**  B. Free text ***(Table S1)*** and No **(3%)**, Yes **(69%)**, Unsure **(28%)**  C. a) The subject still needs to the on the scanner **(23%)**  b) Within 1 hour of acquisition **(13%)**  c) Within 24 hours of acquisition **(44%)**  d) Before the subject's next visit **(20%)** |
| **Q2**   1. **In regards to sensitivity and specificity, what percentage of change is significant enough to change practice? How should this be defined?** 2. **Who should be involved in the decision to change practice?** 3. **Should change in clinical practice be for all patients or selected cohorts?** | A. a) 1-2% **(7%)**; b) 2-10% **(14%)**; c) 10-30% **(53%)**; d) 30-50% **(12%)** e) over 50% **(14%)** and free text ***(Table S1)***.  B. Multiple answers possible a) Clinicians **(97%)**; b) Hospital Managers **(62%)**; c) Radiographers **(71%)**; d) Physicists **(75%)**; e) Other **(23%)**  C. a) All Patients **(8%)**; b) Selected Cohorts **(37%)**; c) Unsure **(55%)** |
| **Q3**   1. **Does "validation" mean something different to a physicist and a clinician?**   **How much do you agree with the following statements.**   1. **"Validation is knowing an imaging biomarker's repeatability and reproducibility."** 2. **"Validation is knowing the accuracy and precision of the measurement."** 3. **"Validation is making a clinician happy to use your imaging biomarker."** 4. **"Validation is knowing how well we can measure an imaging biomarker."** 5. **At what point do you consider something validated?** 6. **How do we incentivise transparency and reproducibility?**   **How much do you agree with the following statement.**   1. **"Funders need to mandate sharing of code."** 2. **"Funders need to mandate sharing of data."** 3. **"Publishers need to mandate sharing of code."** 4. **"Publishers need to mandate sharing of data."** 5. **"Institutions need to provide knowledge support for code sharing."** 6. **"Institutions need to provide knowledge support for data sharing."** | Ai. a) Strongly Agree **(22%)**; b) Agree **(46%)**; c) Neither agree or disagree **(4%)**; d) Disagree **(9%)**; e) Strongly Disagree **(3%)**  Aii. a) Strongly Agree **(14%)**; b) Agree **(48%)**; c) Neither agree or disagree **(9%)**; d) Disagree **(10%)**; e) Strongly Disagree **(1%)**  Aiii. a) Strongly Agree **(4%)**; b) Agree **(22%)**; c) Neither agree or disagree **(14%)**; d) Disagree **(30%)**; e) Strongly Disagree **(13%)**  Aiv. a) Strongly Agree **(19%)**; b) Agree **(33%)**; c) Neither agree or disagree **(22%)**; d) Disagree **(9%)**; e) Strongly Disagree **(3%)**  B. Free text ***(Table S1)***.  Ci. a) Strongly Agree **(23%)**; b) Agree **(43%)**; c) Neither agree or disagree **(21%)**; d) Disagree **(9%)**; e) Strongly Disagree **(4%)**  Cii. a) Strongly Agree **(12%)**; b) Agree **(44%)**; c) Neither agree or disagree **(28%)**; d) Disagree **(11%)**; e) Strongly Disagree **(5%)**  Ciii. a) Strongly Agree **(28%)**; b) Agree **(26%)**; c) Neither agree or disagree **(26%)**; d) Disagree **(14%)**; e) Strongly Disagree **(5%)**  Civ. a) Strongly Agree **(14%)**; b) Agree **(33%)**; c) Neither agree or disagree **(32%)**; d) Disagree **(14%)**; e) Strongly Disagree **(7%)**  Cv. a) Strongly Agree **(50%)**; b) Agree **(41%)**; c) Neither agree or disagree **(7%)**; d) Disagree **(2%)**; e) Strongly Disagree **(0%)**  Cvi. a) Strongly Agree **(53%)**; b) Agree **(35%)**; c) Neither agree or disagree **(11%)**; d) Disagree **(2%)**; e) Strongly Disagree **(0%)** |
| **Q4**   1. **It is known that vendors will find a method less attractive if a method requires a phantom, so when should we use a phantom? Should we use phantoms to standardise data acquisition for every sequence?** 2. **Do you agree with a goal-orientated approach to quality?** 3. **Should we standardise, harmonise or optimise pulse sequences in multi-centre studies?** | A. Free text ***(Table S1)*** and No **(48%)** /Yes **(5%)** /Unsure **(43%)**  B. No **(0%)** /Yes **(52%)** /Unsure **(48%)**  C. a) Standardise **(24%)**; b) Harmonise **(42%)**; c) Optimise **(11%)**; d) Unsure **(24%)** |
| **Q5**   1. **i. At what point should we be sharing code?**   **ii. At what point should we be sharing data?**   1. **How do we balance the need for the subject's privacy against the value of sharing data? i.e. how much can we anonymise without losing valuable information and how do we get consent for making data public?** 2. **Techniques generally require buy-in from scanner manufacturers to become part of their product in order to change clinical practice. How do we balance the need for researchers to protect the IP to allow this to happen, against the value of sharing code and data publicly?** | Ai. a) From the very beginning of a project **(2%)**; b) At the point of publication **(73%)**; c) Once the method is established **(25%)**; d) Never **(0%)**  Aii. a) From the very beginning of a project **(5%)**; b) At the point of publication **(71%)**; c) Once the method is established **(18%)** d) Never **(5%)**  B. Free text ***(Table S1)***  C. Free text ***(Table S1)*** |
| **Q6**   1. **Does a Quality Management System (QMS) exist in your place of work?** 2. **Do you use it?** 3. **How do we incentivise the use of a QMS?** | A. No **(20%),** Yes **(46%),** Unsure **(33%)**  B. No **(50%)**, Yes (**34%)**, Unsure **(16%)**  C. Free text ***(Table S1)*** |
| **Q7**   1. **As an early career researcher, what's the best way to contact clinicians?** 2. **How do we integrate our MR development with PACs systems?** 3. **How do we find out if a patient group can tolerate the imaging method?** | A. Free text ***(Table S1)***  B. Free text ***(Table S1)***  C. Free text ***(Table S1)*** |

**Table 1** Survey questions (column 1) generated from the questions posed at the roundtable discussions at the BIC-ISMRM workshop. Response type for each question is given in column 2 along with the percentage of participants who gave that answer. Free text answers for each question can be found in Table S1 of the supplementary material. Survey found: https://redcap.slms.ucl.ac.uk/surveys/index.php?s=5PdwR6JJdLvK5SfT. Question 3: Data are presented for those in a clinical role (clinicians, radiographers and imaging scientists in a clinical role) and those in a research role (imaging scientist in a research role). Those describing their role as “other” were not included.
